# Anterior eye parameters and lens thickness measured by an intraoperative OCT and a swept-source OCT: comparison of hyperopic, emmetropic and myopic eyes

**DOI:** 10.1038/s41433-023-02506-y

**Published:** 2023-04-19

**Authors:** Michael Müller, Charlotte Wortmann, Julia Paul, Katarzyna Pawlowicz, Eva Hemkeppler, Thomas Kohnen, Myriam Böhm

**Affiliations:** 1 Department of Ophthalmology, Goethe-University, Frankfurt am Main, Germany; 2 Department of Ophthalmology, St. Franziskus Hospital, Münster, Germany; 3 Harvard Medical School, Boston, MA USA

**Keywords:** Outcomes research, Quality of life

## Abstract

**Purpose:**

To evaluate if anterior chamber depth (ACD) and lens thickness (LT) measured by two different devices are affected by different eye lengths.

**Methods:**

ACD and LT of 251 eyes (44 hyperopic, 60 myopic, 147 emmetropic) of 173 patients received with an iOCT-guided femtosecond laser-assisted lens surgery (FLACS) and the IOL Master 700 were compared.

**Results:**

ACD measured with the IOL Master 700 was −0.026 ± 0.125 mm smaller (*p* = 0.001) than that with the iOCT for all eye-groups (hyperopic: *p* = 0.601, emmetropic: *p* = 0.003; myopic: *p* = 0.094). However, differences in all groups were not clinically relevant. LT measurements (all eyes: −0.0642 ± 0.0504 mm) shows a statistically significant difference in all evaluated groups (*p* < 0.001). Only myopic eyes showed a clinically relevant difference in LT.

**Conclusion:**

The two devices show no clinically relevant differences in the eye-length groups (myopic, emmetropic, and hyperopic) for all ACD measurements. LT data shows a clinically relevant difference only for the group of myopic eyes.

## Introduction

With increasing quality expectations of patients to the refractive result of cataract surgery, the accuracy of preoperative measurements of eye parameters to calculate the power of the implanted IOL has become more and more important [1].

The IOL Master (Zeiss Meditec, Jena, Germany) is the mostly used device to measure anterior eye parameters and has shown a high repeatability and agreement in comparison with other devices [2]. While IOL calculation is often of high accuracy in eyes with normal length (between 22 and 26 mm), the situation in hyperopic (<22 mm) and myopic (>26 mm) eyes is different [3, 4]. Here the results in IOL calculation are often more difficult and the postop refraction is less precise. Many different formulas for an optimized IOL calculation have been developed. All of them using different anterior chamber measurements [5–7]. With the introduction of the femtosecond laser technology incorporating intraoperative OCT measurements a new intraoperative possibility for biometry has risen. The minimal invasive surgery technique aims to minimize the intraoperative strain and to maximize the refractive outcome. Based on this, femtosecond laser-assisted cataract surgery (FLACS) gets common to prevent intra- or postoperative complications [8–10]. During FLACS the laser needs precise positioning-data in order to perform the corneal incisions, the capsulotomy [11] and the lens fragmentation. Therefore, just in time before the laser treatment begins, the eye must be measured by an intraoperative OCT (iOCT): an interface docks on the patients´ eye to fix its position while the laser is working [12]. The femtosecond laser comprises an intraoperative OCT of the anterior segment to guide its work.

Recently, Böhm et al. [13] published a paper where they compared the quality of preoperative data of central corneal thickness (CCT), ACD and LT with intraoperative data. They used three different measurement devices: IOL Master 700 (Zeiss Meditec, Jena, Germany), Pentacam AXL (Oculus, Wetzlar, Germany) and iOCT (LenSx, Alcon, Fort Worth, TX, USA). The study revealed only small differences between the devices: only the ACD comparison between the iOCT and the Pentacam AXL showed a clinically relevant difference. Between the IOL Master 700 and the intraoperative iOCT no clinically relevant differences were found.

This current study investigates if different axial eye lengths (in hyperopic, emmetropic or myopic eyes) have an impact on the accuracy of anterior segment parameters, especially ACD and LT, gauged by the IOL Master 700 and the iOCT of the LenSx.

## Materials and methods

This retrospective study included patients, who underwent FLACS between 2016 and 2021 in the Department of Ophthalmology, Goethe University, Frankfurt, Germany. The study was approved by the local ethics committee of the Frankfurt University (Registration No. 409/17) and is in accordance with the Helsinki Declaration. All procedures were performed by the same surgeon (TK) with the LenSx femtosecond laser using the integrated intraoperative OCT (iOCT). All patients received additional anterior segment measurements and a biometry with the IOL Master 700 before surgery to select the required IOL power. ACD and LT measurements were compared between the two devices with regard to differences in the data accuracy in eyes with variable axial length (hyperopic eyes with an AL < 22 mm, emmetropic eyes with an AL between 22.1 and 26 mm and myopic eyes with more than 26 mm) using Bland–Altman method. BIAS [epsilon Verlag, Version 11.11, Germany] and SPSS [IBM, NY, USA] were used for statistical calculations (see below).

### Enrollment of participants

The data of FLACS patients were included for analysis from January 2016 to May 2021. Inclusion criteria were complete measurements of iOCT and IOL Master 700. Patients were excluded from the data analysis in case of corneal pathologies, previous eye surgery or eye trauma that may affect the measurements or if one of the measurements was in a poor quality.

Two hundred fifty-one eyes of 173 patients were enrolled in this retrospective study. Depending on the axial length measured with IOL Master 700 the patients were categorized as hyperopic (axial length < 22 mm), emmetropic (axial length 22–26 mm) or myopic (axial length >26 mm).

### Examinations

#### Intraoperative OCT and femtosecond laser procedure

During FLACS, the interface of the femtosecond laser (LenSx) docks to the patient’s eye using a vacuum. When a good docking is achieved the implemented iOCT performs a one-dimensional picture on the PC-screen where the whole anterior part of the eye from the corneal surface to the posterior part of the eye lens is visible. This scan allows precise planning of the corneal incisions, capsulorhexis and lens fragmentation. All iOCT images were saved and then used for further measurements.

All relevant parameters were measured in a defined sequence using the image processing program FIJI (version 2.0.0 based on IMAGEJ) [14] by two trained persons. A larger number of the iOCT pictures were double checked to ensure reproducibility. Measurements of ACD and LT were taken three times to ensure a high data quality in picture analysis. Here an intraclass-correlation coefficient (ICR) of at least 0.99 was found. If the iOCT-picture quality was poor, this patient’s eye was excluded.

#### IOL Master 700

Before surgery all patients received a complete eye examination including an IOL Master 700 measurement. The IOL Master 700 measures the biometry of the eye and all relevant information for the calculation of the IOL power (keratometry, cornea white to white (WTW), ACD, LT). The IOL Master 700 uses a laser source with a tuneable wavelength of 1055 nm to scan the eye and to additionally measure the posterior surface of the cornea. This swept-source technology allows a higher image quality leading to more accurate results. Further, the IOL Master 700 includes a patented Cornea-to-Retina-Scan which allows a longitudinal scan through the eye to identify untypical geometrics.

#### Statistical analysis

Statistical analysis was performed using BIAS 11. 11 for Windows and Microsoft Excel 2007. Considering a standard deviation (SD) for the measurement difference between IOL Master 700 and the iOCT of 0.14, derived from the data of Böhm et al. [13], a maximal difference of ½ SD was assumed to be clinically significant between the device measurements (Cohen’s *d* = 0.5: medium effect size). Based on this assumption a total of 40 eyes per group were required for a significance level (*α*) of 0.05 and a test power of 0.80 (BiAS for Windows, Version 11.11).

Biometric parameters are presented as mean ± standard deviation (SD) (*t*-Test for parametric and Wilcoxon-Test for non-parametric data). To compare the two devices regarding ACD and LT the Bland–Altman method was used. A *p* value less than 0.05 was considered as statistically significant. To check the clinical relevance between two devices, a clinically relevant difference was defined based on a meta- analysis of Rozema et al. [15] for the external ACD of ±0.085 mm and for the lens thickness ±0.085 mm, which was transferred since the lens has a comparable size as the ACD. If the 95% confidence interval of the mean difference is within the defined range, here the above mentioned ± 0.085 mm, the two devices will be seen as clinically equivalent.

Additionally, after the data acquisition, a power calculation of the Bland–Altman’s method comparison between IOL Master 700 and the LenSx iOCT showed a statistical power of 0.955.

To examine the possible problem of inter-collinearity between the eyes of one patient a further analysis was performed only including one eye per patient. Here, the right or left eye was randomly selected.

## Results

All data is described in detail in Tables 1–5. The statistic for just one eye per patient showed similar results compared to all existing eyes (Tables 3 and 4).

**Table 1:** Baseline characteristics of study participants.

**Each** eye per patient					
		All eyes	Hyperopic	Emmetropic	Myopic
	*N*	251	44	147	60
Eye [right]		124 (49.4%)	23 (52.3%)	70 (47.6%)	31 (51.7%)
Just **one** eye per patient					
		All eyes	Hyperopic	Emmetropic	Myopic
	*N*	173	31	98	44
Sex [female]		82 (47.4%)	17 (54.8%)	43 (43.9%)	22 (50%)
Eye [right]		82 (47.4%)	18 (58.1%)	42 (42.9%)	22 (50%)
Age [years]	Mean	63.7	57.7	67.9	58.1
	±SD	12.850	12.740	12.340	10.120
	Min.	23	23	25	30
	Max.	89	81	89	78
	Median	66	56	71	57

*N* numbers, *SD* standard deviation, *min* minimum, *max* maximum.

**Table 2:** ACD and LT data of all groups for the two devices: 700 vs. iOCT.

	700	iOCT	700	iOCT	700	iOCT	700	iOCT
	All eyes	All eyes	Hyperopic	Hyperopic	Emmetropic	Emmetropic	Myopic	Myopic
	**Each** eye per patient							
ACD								
Mean	3.16	3.19	2.83	2.84	3.16	3.19	3.41	3.43
± SD	0.393	0.376	0.394	0.362	0.369	0.351	0.245	0.218
Min.	1.85	2.01	1.85	2.09	2.04	2.01	2.87	2.90
Max.	4.00	3.96	3.48	3.49	4.00	3.96	3.99	3.78
Median	3.17	3.21	2.88	2.88	3.14	3.18	3.44	3.47
*N*	251	251	44	44	147	147	60	60
	Just **one** eye per patient							
ACD								
Mean	3.17	3.19	2.84	2.84	3.16	3.19	3.41	3.43
± SD	0.402	0.387	0.383	0.355	0.385	0.372	0.267	0.223
Min.	1.85	2.01	1.85	2.09	2.04	2.01	2.87	2.90
Max.	4.00	3.96	3.48	3.47	4.00	3.96	3.99	3.76
Median	3.17	3.22	2.86	2.90	3.15	3.21	3.42	3.49
*N*	173	173	31	31	98	98	44	44
	**Each** eye per patient							
LT								
Mean	4.49	4.55	4.58	4.63	4.49	4.55	4.41	4.50
± SD	0.374	0.373	0.382	0.389	0.404	0.400	0.623	0.274
Min.	3.43	3.49	3.77	3.79	3.43	3.49	3.74	3.84
Max.	5.65	5.69	5.26	5.32	5.65	5.69	4.98	5.06
Median	4.49	4.57	4.62	4.71	4.52	4.61	4.39	4.53
* N*	251	251	44	44	147	147	60	60
	Just **one** eye per patient							
LT								
Mean	4.50	4.56	4.58	4.62	4,51	4,57	4.41	4.50
± SD	0.393	0.390	0.404	0.416	0,423	0,418	0.298	0.295
Min.	3.43	3.49	3.77	3.79	3,43	3,49	3.74	3.84
Max.	5.65	5.69	5.26	5.32	5,65	5,69	4.98	5.06
Median	4.49	4.57	4.65	4.74	4,54	4,62	4.37	4.51
* N*	173	173	31	31	98	98	44	44

*ACD* anterior chamber depth, *LT* length thicknessm *700* IOL Master 700, *iOCT* intraoperative OCT of the LenSx, *N* numbers, *SD* standard deviation, *min* minimum, *max* maximum.

**Table 3:** P-data of the statistical comparison of mean ± SD (*t*-Test) for the parameter: age, ACD and LT.

	Just **one** eye per patient			
Age	vs. hyperopia	vs. myopia		
Emmetropia	**<0.0001**	**<0.0001**		
Hyperopia		0.9021		
**IOL Master 700:**				
	Just **one** eye per patient		**Each** eye per patient	
ACD	vs. hyperopia	vs. myopia	vs. hyperopia	vs. myopia
Emmetropia	**<0.0001**	**<0.0003**	**<0.0001**	**<0.0001**
Hyperopia		**<0.0001**		**<0.0001**
LT	vs. hyperopia	vs. myopia	vs. hyperopia	vs. myopia
Emmetropia	0.41200	0.10490	0.18582	0.11183
Hyperopia		**0.04897**		**0.01472**

*ACD* anterior chamber depth, *LT* lens thickness, *SD* standard deviation.

Bold values denote statistically significant results.

**Table 4:** Bland–Altman statistics between IOL Master 700 and iOCT for the ACD- and LT-parameter.

		*N*	Mean difference ± SD	Test of device difference	CI for mean difference
		**Each** eye per patient			
ACD	All eyes	251	−0.0258 ± 0.1246	**0.00119**	−0.0413/−0.0103
	Hyperopic	44	−0.0118 ± 0.1487	0.60078	−0.057/0.0334
	Emmetropic	147	−0.0294 ± 0.1176	**0.00289**	−0.0486/−0.0102
	Myopic	60	−0.0272 ± 0.1235	0.09369	−0.0591/0.0047
LT	All Eyes	251	−0.0643 ± 0.0549	**<0.0001**	−0.0712/−0.0575
	Hyperopic	44	−0.0491 ± 0.0397	**<0.0001**	−0.0612/−0.037
	Emmetropic	147	−0.0583 ± 0.0391	**<0.0001**	−0.0647/−0.0519
	Myopic	60	−0.0903 ± 0.083	**<0.0001**	**−0.1118**/**−0.0689**
		Just **one** eye per patient			
ACD	All eyes	173	−0.0210 ± 0.1317	**0.03760**	−0.0407/−0.0012
	Hyperopic	31	−0.0023 ± 0.153	0.93504	−0.0584/0.0538
	Emmetropic	98	−0.0246 ± 0.1268	0.05781	−0.05/0.0008
	Myopic	44	−0.0261 ± 0.1284	0.18399	−0.0652/0.0129
LT	All eyes	173	−0.0642 ± 0.0504	**<0.0001**	−0.0718/−0.0567
	Hyperopic	31	−0.0452 ± 0.0424	**<0.0001**	−0.0607/−0.0296
	Emmetropic	98	−0.0588 ± 0.0381	**<0.0001**	−0.0664/−0.0511
	Myopic	44	−0.0898 ± 0,068	**<0.0001**	**−0.1105**/**−0.0691**

*ACD* anterior chamber depth, *LT* lens thickness, *N* numbers, *SD* standard deviation, *CI* confidence interval.

Bold values denote statistically significant results.

**Table 5:** Statistical and clinical relevance of the device comparison by Bland–Altman method.

700 vs. iOCT		Each eye per patient		Just one eye per patient	
		Statistical relevance	Clinical relevance	Statistical relevance	Clinical relevance
		*P*		*P*	
ACD	All eyes	**0.0012**	-	**0.0376**	-
	Hyperopic	0.6008	-	0.9350	-
	Emmetropic	**0.0029**	-	0.0578	-
	Myopic	0.0937	-	0.1840	-
LT	All eyes	**<0.0001**	-	**<0.0001**	-
	Hyperopic	**<0.0001**	-	**<0.0001**	-
	Emmetropic	**<0.0001**	-	**<0.0001**	-
	Myopic	**<0.0001**	**+**	**<0.0001**	**+**

*700* IOL Master 700, *iOCT* intraoperative OCT of the LenSx femtosecond laser, *ACD* anterior chamber depth, *LT* lens thickness.

Bold values denote statistically significant results.

Briefly, in this retrospective study 251 eyes (47.4% right eyes) of 173 patients (47.4% female) were included for analysis. The mean age of all patients was 63.7 (± 12.9) years, median 66 years, range 23–89 years.

The hyperopic group contains 44 eyes, the emmetropic group 147 eyes and the myopic group 60 eyes. The mean and median age of the hyperopic and myopic group was significantly lower than in the emmetropic group. It must be taken into account that people with larger hyperopia or myopia have more psychological strain to alter their refractive situation and are mostly rather willing to let perform a clear lens exchange (Table 1).

The IOL Master data showed, as to expect clinically, that the mean ACD was statistically significant smaller in the hyperopic group than in the emmetropic group or in the myopic group. Regarding the mean of the LT data only a statistically significant difference between the hyperopic and myopic group was seen (Tables 2 and 3).

### Comparison of the two devices

For all 251 eyes together, without regarding the axial eye-length, for ACD the IOL Master 700 measures 3.16 ± 0.393 mm and the iOCT 3.19 ± 0.376 mm. For LT the IOL Master 700 measured 4.49 ± 0.374 mm and the iOCT 4.55 ± 0.373 mm (Table 2).

For ACD between the devices there was a statistically significant difference when all eyes were analyzed. Looking at the subgroups, there was a significant difference for the emmetropic eyes group (*p* = 0.003) respectively a trend toward significance (*P* = 0.058), when only one eye per patient is included. Regarding the LT between the two devices there was for all eyes together and for all subgroups a statistically significant difference.

The Bland–Altman (BA) statistics for ACD showed a mean difference of −0.0258 ± 0.1246 mm and for LT a mean difference of −0.0643 ± 0.0549 mm, indicating that the iOCT measures the ACD and LT a little longer than the IOL Master 700. For all eyes together, without attending subgroups, neither for ACD nor LT, there was a statistically but no clinically relevant difference between the data of the two devices (Tables 4 and 5).

### Comparison of parameters in the hyperopic group

In this group with short axial eye-length for ACD the IOL Master 700 measured 2.83 ± 0.394 mm and the iOCT 2.84 ± 0.362 mm. For LT the IOL Master data was 4.58 ± 0.382 mm and for the iOCT 4.63 ± 0.389 mm (Table 2).

In BA-analysis the mean difference between IOL Master and iOCT for ACD was −0.0118 ± 0.1478 mm and for LT −0.0491 ± 0.0397 mm, meaning that the iOCT measures higher values for both parameters, thus deeper ACD and thicker lenses. For ACD there was neither a statistically nor a clinically relevant difference and for LT there was only a statistically but no clinically relevant difference between the two devices (Tables 4 and 5).

### Comparison of parameters in the emmetropic group

In this group with normal axial eye-length for ACD the IOL Master 700 measured 3.16 ± 0.369 mm and the iOCT 3.19 ± 0.351 mm. For LT the IOL Master data determined 4.49 ± 0.404 mm and the iOCT 4.55 ± 0.400 mm (Table 2).

In BA-analysis the mean difference between IOL Master and iOCT for ACD was −0.0294 ± 0.1176 mm and for LT −0.0583 ± 0.0391 mm, meaning the iOCT measured higher values for both parameters, thus a deeper ACD and thicker lenses. For ACD there was a statistically significant difference when all eyes were analyzed and a trend towards significance only when one eye per patient was considered. For all and just one eye per patient no clinically relevant difference was found. For LT there was only a statistically but no clinically relevant difference between the two devices (Tables 4 and 5).

### Comparison of parameters in the myopic group

In this group with long axial eye-length for ACD the IOL Master 700 measured 3.41 ± 0.245 mm and the iOCT 3.43 ± 0.218 mm. For LT the IOL Master showed values of 4.41 ± 0.623 mm and the iOCT of 4.50 ± 0.274 mm (Table 2).

In BA-analysis the mean difference between IOL Master and iOCT for ACD was −0.0272 ± 0.1235 mm (−0.0261 ± 0.1284 mm) and for LT −0.0903 ± 0.083 mm (−0.0898 ± 0.068 mm), meaning that the iOCT measured higher values, thus deeper ACD and thicker lenses. For ACD there was neither a statistically significant nor a clinically relevant difference. However, in myopic eyes group, for LT there was a statistically significant and a clinically relevant difference between the two devices (Tables 4 and 5).

## Discussion

The expectations on the quality and refractive results of cataract surgeries are rising steadily. This leads to the continuous development and improvement of more precise measuring techniques of the anterior eye parameters (keratometry on the anterior and posterior surface of the cornea, ACD, LT and biometry) like IOL Master 700, Pentacam AXL or intraoperative OCTs and to safer surgery methods like FLACS.

Although the currently used techniques of biometry machines (interferometry, swept-source OCT, Scheimpflug photography and iOCT) show a high measurement quality, differences between the devices are known and especially the situation with shorter or longer eyes is challenging [16–19]. Beyond that, the expectations of the accuracy of IOL power calculation formulas, especially in longer and shorter axial eye lengths, are increasing [5, 20–23]. In consideration of the fact that in clinical practice, mostly “only” one device will be used, it is useful to be aware of possible measurement differences. Recently, it was shown in a study comparing three devices (IOL Master 700, Pentacam AXL and the LenSx iOCT) that in the comparison of the IOL Master 700 vs. the iOCT for the ACD (0.011 ± 0.126, *P* = 0.389) and LT (−0.051 ± 0.089, *P* < 0.001) parameter no clinically relevant differences and only statistically significant differences for the LT were found [13].

This study investigates possible measurement differences of the anterior chamber depth (ACD) and lens thickness (LT) between an iOCT of the LenSx femtosecond laser and a swept-source OCT (IOL Master 700) with special attention to measurement differences that might exist between shorter and longer eyes. To our knowledge this is the first study where these biometry devices are compared to each other differentiating between hyperopic, emmetropic and myopic eyes.

The common demographic data is displayed in Table 1. Looking at the age distribution of the groups with different axial eye lengths, we found that the hyperopic and myopic group was normally distributed, whereas the emmetropic group was not. The mean and median age of the hyperopic and myopic group was significantly lower than in the emmetropic group. Here we must take into account that people with larger hyperopia or myopia have more psychological strain to alter their refractive situation and are earlier willing to let perform a clear lens exchange. Regarding the research question of our study (measuring value comparison), the inhomogeneous age distribution between the subgroups (hyperopic, emmetropic or myopic) does not play a role, because the same eye was measured with two different devices to analyze measurement differences.

A consideration of the frequently used data of the IOL Master data showed typical results for ACD, where the mean was (statistically significant) smaller in the hyperopic group than in the emmetropic group or in the myopic group. The LT data only revealed a statistically significant difference between the hyperopic and myopic group (Tables 2 and 3).

First, analyzing all eyes together without considering the different axial eye lengths, we find that for ACD the IOL Master 700 measures 3.16 ± 0.393 mm (3.17 ± 0.402 mm) and the iOCT 3.19 ± 0.376 mm (3.19 ± 0.387 mm). For LT the IOL Master 700 measured 4.49 ± 0.374 mm (4.50 ± 0.393 mm) and the iOCT 4.55 ± 0.373 mm (4.56 ± 0.390 mm) (Table 2). The Bland- Altman analysis of all eyes together, showed a mean difference between the devices of −0.0258 ± 0.1246 mm (*P* = 0.00119) ((−0.0210 ± 0.1317 mm) (*P* = 0.03760)) for the ACD and −0.0643 ± 0.0549 mm (*P* < 0.0001) ((−0.0642 ± 0.0504 mm) (*P* < 0.0001)) for the LT. Comparing our data with results of Böhm et al. [13], who reported a mean difference for ACD of 0.011 ± 0.126 mm (*P* = 0.3889) and for LT −0.051 ± 0.089 mm (*P* < 0.001) between the same devices, we found similar results for the LT-parameter. However, in our study the iOCT measures a deeper ACD than the IOL Master (−0.0258 ± 0.1246 (*P* < 0,01)). In 2021 a study of Tana-Sanz et al. [24] compared the intraoperative SD-OCT of the Catalys femtosecond laser with the IOL Master 700 and found statistically significant differences for ACD, LT and other parameters. The mean difference between the devices for LT ranged from −0.02 to −0.08 mm, and can be interpreted as “quite similar”, and is in line with our data, except our finding of a clinically relevant difference for LT in myopic eyes. For the ACD parameter Tana-Sanz et al. mentioned a larger difference between the intraoperative SD-OCT and the SS-OCT in the IOL Master 700.

In principle, there is no consistent explanation for the measuring difference. It could be presumed, that the suction process during FLACS and the different measuring positions of the devices (sitting position at the IOL Master and a lying patient at the iOCT) could be effective. Pahlitzsch et al. [25] showed that the ACD in FLACS is statistically significantly larger than the ACD in “normal” phacoemulsification (4.05 vs. 3.77 mm, *p* = 0.023). Also, in a prior study by Sel et al. [26], where the IOL Master 700 was compared to other devices like the Pentacam AXL, the ACD was measured significantly higher (*p* < 0.001) by the other devices than by the IOL Master. The difference between the two studies is off the range of clinical relevance. The finding that FLACS is leading to a higher ACD could be explained by an effect of the suction process. Additionally, the suction process could minimize the scleral traction on the zonula fibers, having an effect on the lens leading to a rounding of the lens, thus enlarge the LT.

### Differences between the subgroups (hyperopia, emmetropia and myopia)

A consideration of the clinically often used IOL Master showed typical results for the different eye-length groups regarding ACD and LT (IOL Master 700 data in Table 2): as generally recognized, the ACD was smaller in hyperopic eyes in contrast to emmetropic or myopic eyes. In relation to LT, there was no significant difference between emmetropic eyes vs. hyperopic or myopic eyes, but a slightly statistically significance between hyperopic vs. myopic eyes, where LT in hyperopic eyes is showing a trend towards higher measurements (Table 3).

The Bland–Altman analysis (Table 4) showed for both parameters (ACD and LT), independent of axial eye-length, for the IOL Master 700 that it measures flatter than the iOCT. For ACD the mean difference between the two devices was statistically significant only for all eyes together (*P* = 0.00119) (*P* = 0.03760), whereas in the subgroups, only a statistically significance for all emmetropic eyes together and, if just one eye per patient was analyzed, a trend towards significance was seen. For LT there was a statistically significance for all groups, but a clinical relevance was only found for the myopic eye subgroup (Table 5).

Here, for myopic eyes there was a clinically relevant difference between the measurement data of the IOL Master 700 and the iOCT, where the iOCT provides thicker lenses. Emanating from these thicker measured lenses with the iOCT, this, in FLACS, should receive attention: if the LT is really smaller than measured by the iOCT, the surgeon has to be aware of the minimal chosen distance for the femtosecond laser exposition to the posterior capsule in order not to endanger their integrity.

Although most of the measurement differences are small and not clinically relevant for IOL calculations, they could become relevant regarding ICL calculation and size selection.

The main limitation of this study is its retrospective nature. However, this limitation is not that relevant due to the fact that only healthy eyes, without a prior history of eye surgeries, traumata or other (severe) ocular pathologies, were used for analysis. Secondly, the devices are measuring in different patient positions (sitting / lying) what could impact the shape of the anterior segment of the eye. Furthermore, the iOCT measurements-since they were taken direct at the beginning of the FLACS-could not be repeated, meaning that no statement to the reproducibility is possible. Though, the postoperative evaluation of the iOCT pictures itself showed a high interclass-correlation-coefficient.

Our study shows that iOCT measurements of ACD and LT during FLACs are in the majority of cases very good comparable to IOL Master 700 measurements. Clinically relevant differences were only found in myopic eyes, where the iOCT measures in the mean a larger LT. Here, special care is needed to interpret the iOCT data of the LT in myopic eyes and further investigations are useful to improve the predictability.

If the iOCT data during FLACS would be combined with a biometry device, it could be possible to accomplish also an intraoperative IOL calculation. In an interim time, parallel both devices (iOCT and IOL Master) could be used, given the possibility to double check each other and improve the accuracy of IOL selection. Maybe, someday, we are able to resign the necessity of preoperative biometry and IOL calculation.

## Summary

### What was known before

- Intraoperative OCTs are increasingly used in FLACS. Previous comparisons found only a statistically significant difference for the LT measurement but no clinically relevant differences between the LenSx iOCT and the swept-source OCT (IOL Master 700) regarding the ACD and LT.

### What this study adds

- Compared to the IOL Master 700, the intraoperative OCT is measuring in the mean always larger values for the ACD- and LT-parameter. Although statistically significant for all eyes together, clinically relevant differences were only found for the LT-parameter in myopic eyes.
- It should be critically considered if iOCT measurements could be embedded in an intraoperative IOL calculation.

## Data Availability

The datasets generated and/or analyzed during the current study are available from the corresponding author on reasonable request.
